# Mathematical modeling and numerical simulation technique for selected heavy metal transport in MSW dumpsite

**DOI:** 10.1038/s41598-023-32984-9

**Published:** 2023-04-07

**Authors:** G. A. Usoh, Isiguzo Edwin Ahaneku, E. C. Ugwu, E. O. Sam, D. H. Itam, George Uwadiegwu Alaneme, T. C. Ndamzi

**Affiliations:** 1 Department of Agricultural Engineering, Akwa Ibom State University, Ikot Akpaden, Mkpat Enin, Nigeria; 2 Department of Agricultural and Bioresources Engineering, Michael Okpara University of Agriculture, Umudike, Nigeria; 3 Department of Civil and Environmental Engineering, University of Calabar, Calabar, Cross Rivers State Nigeria; 4 Department of Civil Engineering, Kampala International University, Ggaba Road, Box 20000, Kampala, Uganda; 5 Department of Agricultural and Environmental Engineering, Rivers State University, Port Harcourt, Nigeria

**Keywords:** Mathematics and computing, Civil engineering

## Abstract

The study focused on development of mathematical modeling and numerical simulation technique for selected heavy metal transport in Uyo municipal solid waste dumpsite in Akwa Ibom State to investigate the level in depth to which leachate from the dumpsite extends and the quantity of leachate at various depth of the dumpsite soil. Uyo waste dumpsite is operating open dumping system where provisions are not made for preservation and conservation of soil and water quality, hence, the need for this study. Three monitoring pits within Uyo waste dumpsite were constructed and infiltration runs were measured, and soil samples were collected beside infiltration points from nine designated depths ranging from 0 to 0.9 m for modeling heavy metal transport in the soil. Data collected were subjected to descriptive and inferential statistics while the COMSOL Multiphysics software 6.0 was used to simulate the movement of pollutants in the soil. It was observed that heavy metal contaminant transport in soil of the study area is in the power functional form. The transport of heavy metals in the dumpsite can be described by a power model from linear regression and a numerical model based on finite element. Their validation equations showed that the predicted and the observed concentrations yielded a very high R^2^ value of over 95%. The power model and the COMSOL finite element model show very strong correlation for all selected heavy metals. Findings from the study has identified level in depth to which leachate from the dumpsite extends and the quantity of leachate at various depth of the dumpsite soil which can be accurately predicted using leachate transport model of this study.

## Introduction

Solid waste disposal sites like open dumps represent a significant source of metals released into the environment^1–5^. Soil contaminated by heavy metals from solid waste disposal sites is a serious problem because soils are regarded as the ultimate sink for heavy metals discharged into the environment, as many heavy metals are bound to soils^6^. The soil may be contaminated with heavy metals such as lead, copper, zinc, iron, manganese, chromium and cadmium and these heavy metals in solid waste led to serious problem because they cannot be biodegraded. According to Freeze and Cherry^7^ leachate from a solid waste disposal site generally contain major elements like calcium, magnesium, potassium, nitrogen and ammonia, trace metals like iron, copper, manganese, chromium, nickel, lead and organic compounds like phenols, polyaromatic hydrocarbons, acetone, benzene, toluene and chloroform. According to Ahaneku and Sadiq^8^, heavy metal uptake in agricultural soils is of great concern due to food safety issues and potential health implications. MSW leachate varies widely in composition, contains both dissolved and suspended materials depending on the age of the dumpsite and the type of solid waste. Leachate that escapes from the MSW dumpsite may migrate through the unsaturated zone and eventually reach the groundwater table and then get transported through the saturated zone to a point of discharge (i.e., a pumping well, a stream, a lake, etc.) thereby causing contamination.

Modeling is the process by which scientists represent ideas about the natural world to each other, and then collaboratively make changes to these representations over time in response to new evidence and understandings^9,10^. A model can come in many shapes, sizes, and styles. It is important to emphasize that a model is not the real world but merely a human construct to help us better understand real world systems. In general, all models have an information input, an information processor, and an output of expected results. Models do not just reflect reasoning, they also stimulate new ideas^11,12^. According to Ndirika and Onwualu^13^, model is a representation of the construction and working of some system of interest; model is similar to but simpler than the system it represents; one purpose of a model is to enable the analyst or the researcher to predict the effect of changes to the system. Pachepsky et al.^14^ developed a generalized Richards’ equation to simulate water transport in unsaturated soils. Simulations of water transport in soil are ubiquitous for experiments on water transport in soil horizontal columns, Richards’ equation predicts that volumetric water contents should depend solely on the ratio (distance)/(time)q where q ¼ 0:5: Substantial experimental evidence shows that value of q is significantly less than 0.5 in some cases. Nielsen et al.^15^ related values of q, 0.5 to ‘jerky movements’ of the wetting front, i.e. occurrences of rare large movements. The corresponding mathematical model is a generalized Richards’ equation in which the derivative of water content on time is a fractional one with the order equal or less than one. The equation was first solved numerically and then fitted the solution to data on horizontal water transport. For such systems, Richards’ equation reduces to the mathematical expression presented in Eq. (1).

1 $$ \frac{\partial \theta }{{\partial {\text{t}}}} = { }\frac{\partial }{{\partial {\text{x}}}}\left[ {{\text{D}}\left( \theta \right)\frac{\partial \theta }{{\partial {\text{x}}}}} \right] $$

where $\theta$ is the volumetric soil water content, D is the soil water diffusivity, x is the distance from one of the ends of the column, t is time. Soil bulk density changes and soil water hysteresis are ignored in this formulation^16^. Introduction of the Boltzmann variable transforms Eq. (1) into an ordinary differential equation presented in Eqs. (2), (3).

2 $$ {\uplambda } = { }\frac{{\text{x}}}{{{\text{t}}^{0.5} }} $$

3 $$ - \frac{\lambda }{2}\frac{{{\text{d}}\theta }}{{{\text{d}}\lambda }} = { }\frac{{\text{d}}}{{{\text{d}}\lambda }}\left[ {{\text{D}}\left( \theta \right)\frac{\partial \theta }{{\partial \lambda }}} \right] $$

which has been used to find analytical solutions for soil water flow problems and also to find the dependence of the diffusivity D on soil water content $\theta$^17^. If Eq. (3) is applicable then soil water content is a function of the Boltzmann variable l, and, for the same values of soil water content, one should expect the same values of the Boltzmann variable. Validity of Eq. (3) can be tested with experimental data consisting of observed soil moisture changes during infiltration in horizontal soil columns with initially uniform soil water content. Distances and times at which the same values of water content have been observed must be noted as shown in Eqs. (4), (5).

4 $$\frac{{\mathrm{x}}_{1}}{{{\mathrm{t}}_{1}}^{0.5}}= \frac{{\mathrm{x}}_{2}}{{{\mathrm{t}}_{2}}^{0.5}}=\frac{{\mathrm{x}}_{3}}{{{\mathrm{t}}_{3}}^{0.5}}$$

Meanwhile,

5 $$\mathrm{x}=\mathrm{ A}{\mathrm{t}}^{0.5}$$

where the multiplier A depends only on water content. This equation means that the dependence between log(x) and log(t) plotted in log–log coordinates is linear and the slope of this dependence is 0.5 whereas the intercept depends on the water content. Significant deviations from Eq. (5) have been observed in many published experiments. Gardner and Widtsoe^18^ and Nielsen et al.^9^ recorded the progress of the wetting front in air-dry soil uniformly packed in horizontal columns and a negative pressure head was held at one end of the columns. The largest distance where the wetting front was observed was 50 cm. A linear dependence can be traced in mathematical relationship expressed in Eq. (6).

6 $$\mathrm{logx}=\mathrm{ LogA}+\mathrm{qlogt}$$

As a key to the management of groundwater, Patil and Chore^19^ demonstrated the usefulness of mathematical models in the study of the movement of fluids and contaminants in the subsurface environment. They compared experimental, analytical solution and numerical method in the evaluation of soil and groundwater contaminants transport. Similarly, Islam et al.^20^ applied the governing equation for contaminant transport involving advection–diffusion and using the explicit finite difference method to evaluate groundwater contaminants. Several researchers have studied pollutant migration in solid waste dumpsite. However, very few literatures on application of finite element method to predict contaminant transport exist.

In order to predict reliably and effectively the pollutant contaminant transport in soil layers, it is necessary to develop a comprehensive mathematical modelling of the heavy metal concentration in the soil column, and to design an efficient simulation procedure. In this study, a general approach which combines a mathematical modelling method and Finite Element Method (FEM) simulation is proposed to investigate the concentration of selected heavy metals passing through saturated soil layers. An approach to modelling and computation of the spread of the heavy metal due to the leachate immigration into the soil layers and groundwater at the actual municipal solid waste landfill site was implemented to validate the proposed novel mathematical modeling and FEM-based algorithms using COMSOL Multiphysics, in which the initial value of the selected metal concentrations were calculated by using the data from the experiments which are analyzed from the soil samples collected from the actual municipal solid waste dumpsite. COMSOL Multiphysics is easy, seamless interface between solute transport and other physics fields. It has the ability to modify governing equations, and flexibility in solver selection in the form of direct or fully coupled solutions^21–23^ which makes it unique from other applications. The newly introduced Multiphysics method for modelling the spread of the metal concentration in the leachate immigration in the 2D space of soil layers, and the new procedure for simulating the pollutant transport in the Multiphysics interface are the main contributions of this study.

## Methodology

### Study area

The study was conducted at Uyo Municipal Solid Waste Dumpsite in Uyo Local Government Area, Akwa Ibom State, Nigeria. Uyo, the capital of Akwa Ibom State lies between latitude 4°30″ and 5°30″ N and longitudes 7°30″ and 8°30″ E. Uyo is within the equatorial region characterized by wet and dry seasons. According to Robert^24^, the most outstanding feature of the equatorial climate is its uniformity of temperature throughout the year. Rainfall begins about March and ends around October with a little dry spell called “the August break” occurring in August^25–28^.

With its location within the tropical rainforest and dense population, Uyo, like other major cities in Nigeria generates enormous municipal solid waste which is not adequately managed. The site is used by Environmental Protection and Waste Management Agency for waste disposal. Most of the wastes disposed are domestic and household wastes. This dumpsite is operated as an open dumpsite. The waste dumpsite has function efficiently for about twenty years and there is surface water at the vicinity of the waste dumpsite.

### Collection and analysis of sample

**Figure 1:**
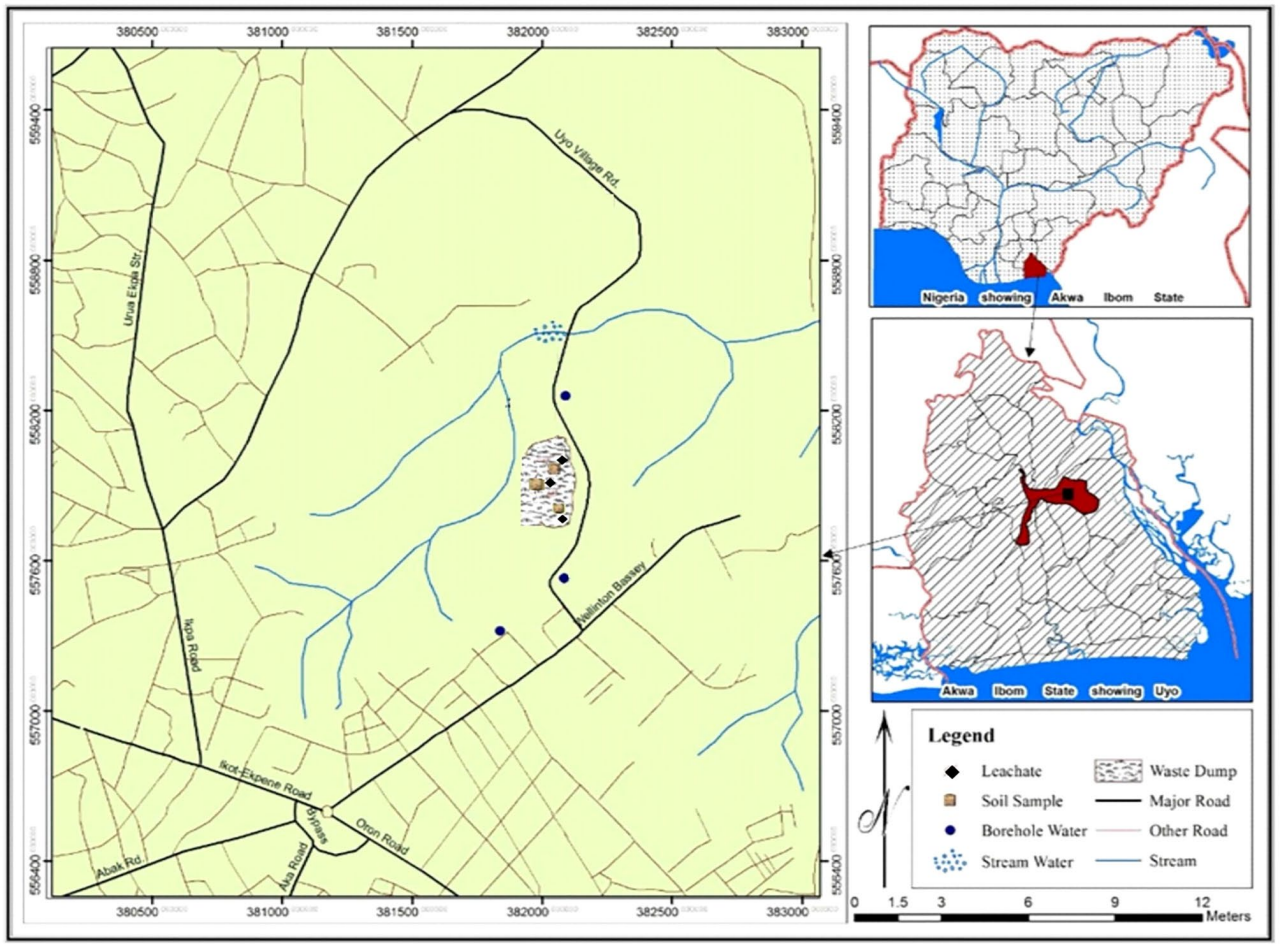
Map of Uyo Local Government Area showing the waste dumpsite and sample collection points (Source: AISMLS, 2020).

Nine core samples were collected at each of the three leachate monitoring stations constructed within the waste dumpsite after infiltration runs were conducted on each of the three spots using synthetic container (Fig. 1). Core samples were collected from the nine designated depth zones with core cylinders measuring 10, 20, 30, 40, 50, 60, 70, 80 and 90 cm in height and 4.5 cm internal diameter for the determination of leachate flux (q). The cylinders were driven into each of the soil by hitting a piece of wood placed on top of the cylinder making sure that the cylinder did not tilt at any point. The soil was excavated from around the cylinder and the soil beneath the cylinder bottom was cut. Excess soil from the cylinder end was trimmed. The bottom of the cylinder was covered with a piece of cloth hold in place with a rubber band and then labeled and taken to laboratory. Heavy metals namely Fe, Cu, Zn, Pb, Mn, Ni and Cd were determined using double beam DW-AA320N Atomic absorption spectrometer. Infiltration tests were made using Gilson HMA-635 double ring infiltrometer apparatus.

### Infiltration measurement

Infiltration runs were carried out beside each leachate monitoring stations. Infiltration tests were made using the double ring infiltrometer method^29^. The infiltrometer, consisting of an outer ring with diameter 50 cm and an inner ring with diameter 30 cm were placed concentrically on the soil surface and carefully driven 10 cm into the soil using a driving plate and impact absorbing hammer. The soil surface within the rings was covered with leaves to protect it from forming the kinetic force of water and to prevent direct splash. Sediment free water was first poured into the outer ring and allowed to infiltrate. Immediately afterwards, water was added to a depth of 15 cm in the inner ring. The outer ring acted as a buffer to discourage lateral flow of the water in the inner ring. Infiltration rate was measured from the inner ring for 2 h using a collaborated float hold in place by a bridge. The intake was recorded and the data was used to determine the infiltration rate of initial and final infiltration rate as well as other infiltration characteristics with soils.

### Computation of sorptivity and transmissivity

Soil water sorptivity (S) which represents the ability of a soil to absorb or desorb water by capillary processes, and transmissivity (A) a measure of the ability of the soil to conduct the flow of water were determined graphically by fitting the approximate Philip^30^ algebraic infiltration equation into the field data. The primary data are measured values of cumulative infiltration *I* expressed in cm, as a function of time. The values represent the total amount of water infiltrated into the soil surface from the beginning of the infiltration test. Philip^30^ showed that the cumulative, one-dimensional infiltration commonly encountered in the field, can easily be expressed as a function of time, I (t), thus:

7 $$\mathrm{I}={\mathrm{St}}^{1/2}+\mathrm{At}$$

where I (cm) is cumulative infiltration at time t (sec.), S (cm sec^−1^) is the soil water Sorptivity obtained as the slope of I versus √t, A (cm sec^−1^) is the soil water transmissivity related to soil’s hydraulic conductivity and is the intercept, and the infiltration rate is:

8 $$\mathrm{I}=\frac{1}{2}{\mathrm{St}}^\frac{1}{2}+\mathrm{A}$$

Subsequently, the parameters *S* and *A* which were obtained graphically were fitted into Eq. (8) to calculate the infiltration rate. The Philip^30^ equation is a mechanistic infiltration model because it is derived from the physically-based water flow equation, i.e. from sound principles of physics. The model was chosen because the parameters *S* and *A* give considerable insight into the hydraulic conditions which prevail in the soil profile prior to and during the infiltration process, and, excluding the development of infiltration inhibiting factors, aid the understanding of the decaying infiltration rate with elapsed time. However, the curve-fitting procedure was adopted which converted the equation into an empirical rather than a physical model.

### Components of the model

There are four major components arising from the different forces that influence leachate and its potential in the soil. These include:

- The matric force: This refers to attraction of leachate to solid particle surfaces and capillary attraction in pore spaces. This force gives rise to the matric potential, M, which is always negative.
- Osmotic force: This refers to attraction of leachate to ions, giving rise to the osmotic or solute potential, o which is always negative too.
- Gravitational force: This refers to attraction of leachate to center of the earth. It is a downward pull on leachate. It gives rise to the gravitational potential, ψg which may be positive or negative depending on elevation of that point relative to a reference elevation. If the point is higher than the reference elevation the ψg is positive but if lower then ψg is negative.
- Pressure of leachate molecules: This gives rise to pressure potential p, which is always positive. Hence, total leachate potential at a point along the soil column (ψ_T_) is given by the formula in Eq. (9):
  9 $${\uppsi }_{\mathrm{T}}={-\uppsi }_{\mathrm{m}}+ {\uppsi }_{\mathrm{p}} + {\uppsi }_{\mathrm{m }}+ \left(-{\uppsi }_{\mathrm{o}}\right)+\left(-{\uppsi }_{\mathrm{g}}\right)$$

where ${\uppsi }_{\mathrm{T}}$ = Total soil leachate potential, ψ_m,_ ψ_p,_ ψ_o_ and ψ_g_ are the matric, pressure, osmotic and gravitational potentials respectively. In terms of head presented in Eq. (10):

10 $${\mathrm{H}}_{\mathrm{T}}={-\mathrm{H}}_{\mathrm{m}}+ {\mathrm{H}}_{\mathrm{p}} + {\mathrm{H}}_{\mathrm{m }}+ \left(-{\mathrm{H}}_{\mathrm{o}}\right)+\left(-{\mathrm{H}}_{\mathrm{g}}\right)$$

where ${\mathrm{H}}_{\mathrm{T}}$ is the total head (cm), ${\mathrm{H}}_{\mathrm{m}}$, ${\mathrm{H}}_{\mathrm{p}}$, ${\mathrm{H}}_{\mathrm{o}}$ and ${\mathrm{H}}_{\mathrm{g}}$ are the matric, pressure, osmotic and gravitational heads respectively.

Generally, leachate is produced on the soil surface but its flow into the soil occurs when there is a difference in total soil leachate potential (TSLP) between the point of production to point of deposition. Hence, the driving force is the gradient (the change) in the total leachate potential between the two points. For instance, considering two points in the soil A and B; at point A, TSLP is higher than at point B. Leachate will then flow from point A to pint B. The driving force is therefore the gradient given as expressed in Eq. (11):

11 $$\mathrm{DF}=\frac{{\uppsi }_{\mathrm{A}}- {\uppsi }_{\mathrm{B}}}{\mathrm{A B}}$$

where $\mathrm{DF}$ is the driving force ${\uppsi }_{\mathrm{A}}\mathrm{ and }{\uppsi }_{\mathrm{B}}$ are the TSLP at points A and B while L_A B_ is the distance between point A and B in the soil. Therefore, leachate flow will always occur in the direction of decreasing TSLP.

### Basic principles of the model

There are three basic principles covering this Soil Leachate Transport Model (SLTM):

i) The driving force of leachate for the transport of leachate from the point of production (soil surface) down the soil is the gradient in the intensity of the components of Total Soil Leachate Potential (TSLP).
ii) Flow of leachate down the soil is directly proportional to the intensity gradient
iii) Flow is in the direction of decreasing intensity

### Mathematical expression of the model

The driving force (DF) is the gradient in total hydraulic head (intensity) between the two points within which flow is occurring. It should be recalled that hydraulic head, H, is the leachate potential ψ, expressed as weight or distance. Hence, Total hydraulic head, H, is defined as presented in Eq. (12):

12 $$\mathrm{H}=\mathrm{h}+\mathrm{ z}$$

where h is the pressure head of water and z is the gravitational head, all expressed in cm.

**Figure 2:**
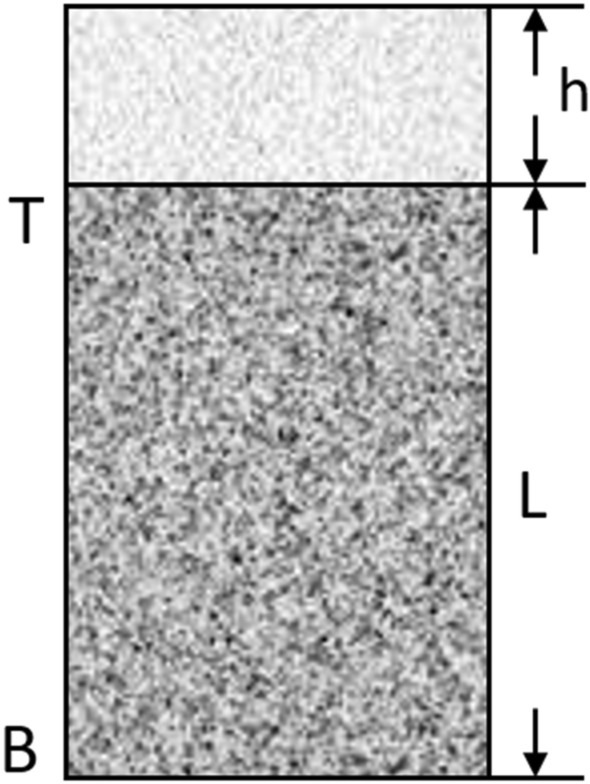
Hypothetical description of soil leachate transport in a unit soil area.

Consider a vertical soil column, L (cm) long with a cross sectional area, A (cm^2^). The top of the column, T is ponded with water to a height of h cm and leachate is just dripping from the bottom, B, of the column as shown in Fig. 2.

The above hypothetical case is used to define the driving force used in this model and other concepts associated with leachate flow in the soil according to the outlined assumptions of this model. Driving force (DF) is the gradient in the total hydraulic head between the top (point T) and the bottom (point B). Total hydraulic gradient therefore is defined by the formula in Eq. (13):

13 $$\frac{\mathrm{\Delta H}}{\mathrm{\Delta X}}= \frac{{\mathrm{H}}_{\mathrm{T}}- {\mathrm{H}}_{\mathrm{B}} }{{\mathrm{X}}_{\mathrm{T }}- {\mathrm{H}}_{\mathrm{B}}}$$

where H_T_ and H_B_ are the total hydraulic heads at T and B respectively and X_T_ and X_B_ are distances at points T and B, respectively. Substituting Eq. (13), into Eq. (12), it becomes the expression in Eq. (14):

14 $$\frac{\mathrm{\Delta H}}{\mathrm{\Delta X}}= \frac{{(\mathrm{h}}_{\mathrm{T}}+ {\mathrm{z}}_{\mathrm{T}})-({\mathrm{h}}_{\mathrm{B}}+{\mathrm{z}}_{\mathrm{B }})}{{\mathrm{X}}_{\mathrm{T }}- {\mathrm{X}}_{\mathrm{B}}}$$

Substituting h_T_ = h cm, Z_T_ = 1 cm, h_B_ = 0 cm, Z_B_ = 0 cm, X_T_ = 0 cm, and X_B_ = − 1 cm into (12) yields the mathematical relationship in Eq. (15):

15 $$\frac{\mathrm{\Delta H}}{\mathrm{\Delta X}}=\frac{(\mathrm{h}+ 1)-(0+0)}{0-(- 1)}= \frac{\mathrm{h}+ 1}{1}$$

Thus, driving force (DF) is expressed in Eq. (16):

16 $$\frac{\mathrm{\Delta H}}{\mathrm{\Delta X}}= \frac{\mathrm{h}+\mathrm{ L}}{\mathrm{L}}$$

Since, leachate is on the top of the column especially when it is much as in during rainfall event, the top T, is therefore saturated and experiencing hydrostatic pressure or positive pressure potential. The height of the leachate above this point is the numerical value of the leachate pressure head (cm) at point T^31^. Since leachate is just dripping at the bottom, it is at atmospheric pressure and therefore at this point is zero. The above applies to unsaturated condition. The second principle was based on the second assumption that the flow of leachate down the soil is directly proportional to the intensity gradient. This was used to model the flux which is defined as the amount of leachate flowing through a unit cross sectional area of the soil per unit time. Considering Fig. 2, flux of leachate between T and B is given by the expression in Eq. (17):

17 $$ {\text{q}} = \frac{{\text{Volume of leachate}}}{{{\text{Area }} \times {\text{ time}}}}{ } = { }\frac{{\text{Q}}}{{{\text{At}}}}{ } = { }\frac{{{\text{cm}}^{3} }}{{{\text{cm}}^{{2{ }}} {\text{sec}}}} $$

But this flux is proportional to the hydraulic head gradient and this is the Darcy’s law of steady state as expressed in Eq. (18)^32^:

18 $$\mathrm{q}= \frac{\mathrm{Q}}{\mathrm{At}} = -\mathrm{K}\frac{\mathrm{\Delta H}}{\mathrm{\Delta X}}$$

where K is the proportionality constant known as hydraulic conductivity. Hence, Eq. (17) is the SLTM which defined amount of leachate flux as the product of the hydraulic conductivity and hydraulic head gradient. The negative sign indicates that the flux is in the direction of decreasing hydraulic head as stated in the third assumption.

According to Eq. (18), it is possible to model leachate transport rates for any head difference over any distance in a given growing medium. The real difficulty, however, is in finding K. According to Allaire et al.^33^, K may be found with a series of formulae according to the structure like in Eqs. (17) and (18) as follows in Eq. (19):

19 $$ {\text{K}} = {\text{ Ksat}}\left( {\frac{{{\text{WEP}}}}{{{\text{TPS}}}}} \right)^{{\text{m}}} $$

where K is unsaturated hydraulic conductivity, Ksat is the saturated hydraulic conductivity, WEP is the leachate fill pores fraction, TPS is the total pores space (total porosity) while m is the constant defined by type of soil structure as follows:

i) Very fine structure: m = 1
ii) Fine structure: m = 2
iii) Medium structure: m = 3
iv) Blocky, Platy, Massive structure: m = 4

The influence of WEP on K is very large as can be seen from the exponential function. The relation WEP over TPS represents the tortuosity of the transport path. The measurement of either Ksat or Kh requires careful pre-treatment of the samples and apparatus so to overcome error in determination of Ksat, Eq. (20) was adopted as used by Allaire et al.^33^ to determine unsaturated hydraulic conductivity directly as follows in Eq. (20):

20 $$\mathrm{I}={\mathrm{C}}_{1}\mathrm{t }+ {\mathrm{C}}_{2}\sqrt{\mathrm{t}}$$

where I is infiltration rate, C_1_ is the transitivity which is the hydraulic conductivity (unsaturated), C_2_is the soil Sorptivity. The numerical solution of Eq. (20) produced the soil leachate transport model of the study area referred to as LEATRAM.

21 $$\mathrm{q}=\mathrm{ C}{\mathrm{x}}^{-\mathrm{n}}$$

where q is the soil leachate flux (cm hr^−1^), C is transmissivity coefficient which depends on the overall soil characteristics, x is the thickness of the soil (cm) representing depth of transport while the negative exponent represent the influence of soil structure called tortuosity, indicating that the flux is in the direction of decreasing hydraulic head as stated in the third principle of the model and n is the non linear exponent which varies with volume of leachate and location of the soil.

### Contaminant transport model

The bulk motion of the fluid and contaminant transport through the soil column by molecular diffusion and mechanical dispersion is negligible. At the same time, generation of loss of mass takes place due to adsorption and biokinetics of the mass dissolved or suspended in the moving water. In this study only heavy metals namely Fe, Cu, Zn, Pb, Mn, Ni, and Cd were modeled. In general, for steady–state water flow condition, the transport terms for heavy metals are as given in Eq. (22):

22 $${\mathrm{J}}_{\mathrm{s}}={\mathrm{J}}_{\mathrm{DL}}+ {\mathrm{J}}_{\mathrm{CL}}$$

where ${\mathrm{J}}_{\mathrm{s}}$ is total heavy metal flux (mg kg^−1^), ${\mathrm{J}}_{\mathrm{DL}}$ is the diffusion flux in the liquid phase and ${\mathrm{J}}_{\mathrm{CL}}$ is the convection flux in the liquid phase. In the case of diffusion in the liquid phase in a porous media, the equation represented by Fick’s law is given as shown in Eq. (23):

23 $${\mathrm{J}}_{\mathrm{DL}}={\mathrm{D}}_{\mathrm{m}}\left(\uptheta \right)+ {\mathrm{J}}_{\mathrm{CL}}$$

where ${\mathrm{D}}_{\mathrm{m}}(\uptheta )$ is the molecular diffusion coefficient. The value of ${\mathrm{D}}_{\mathrm{m}}(\uptheta )$ can be determined through Eq. (23) as given by Kelley^34^ in Eq. (24):

24 $${\mathrm{D}}_{\mathrm{m}}\left(\uptheta \right)={\mathrm{D}}_{\mathrm{OL}}.\mathrm{a}.\mathrm{exp}\left(\mathrm{b\theta }\right)$$

where D_OL_ is the diffusion coefficient in a pure liquid phase and a and b are empirical constants reported by Olsen and Kemper^35^ to be approximately b = 10 and 0.005 < a < 0.01. The convective flux of respective heavy metal is represented as expressed in Eq. (25)^8^:

25 $${\mathrm{J}}_{\mathrm{cL}}={-\mathrm{\theta D}}_{\mathrm{h}}\left(\mathrm{q}\right)+ {\left(\uptheta \right)\frac{{\mathrm{dC}}_{\mathrm{l}}}{\mathrm{dz}}}_{1}+{\mathrm{qC}}_{1}$$

where q is the water flux, and D_h_ (q) is the hydrodynamic dispersion coefficient that describes mixing between large and small pore as the result of local variations in mean water flow velocity. Partitioning the heavy metal between absorbed and solution phases, according to Alemi et al.^36^, adsorption of elements are assumed taken to be nonlinear equilibrium process described by the mathematical relationship in Eq. (26):

26 $${\mathrm{C}}_{\mathrm{s}}={\mathrm{K}}_{\mathrm{s}}{\mathrm{C}}^{\mathrm{n}}$$

where *C*_*s*_ is the concentration of the elements absorbed on the soil (mg k Kg^−1^), Ks is the adsorption coefficient for respective metal (L Kg^−1^), C is the concentration of the element in soil solution (mg k Kg^−1^), n is the nonlinear equilibrium adsorption reaction exponent for respective metals. The total concentration of contaminants (C_T_) contained in the solution and adsorbed phases in a soil volume of one liter is given as presented in Eq. 27:

27 $${\mathrm{C}}_{\mathrm{T}}={\mathrm{\rho C}}_{\mathrm{s}}+\uptheta {\mathrm{C}}_{\mathrm{l}}$$

where ρ is the soil bulk density (g cm^−3^). Substituting Eq. (24) for Cs in Eq. (25) gives the convection–dispersion formula in Eq. (28):

28 $${\mathrm{C}}_{\mathrm{T}}={\mathrm{C}}_{\mathrm{l}}+\left(\uptheta +\uprho {\mathrm{K}}_{\mathrm{s}}\right)$$

Heavy metal transports in soil system occur under non-steady (transient) water flow condition and it varies with depth and time as given in Eq. (29):29

### Numerical solution

**Figure 3:**
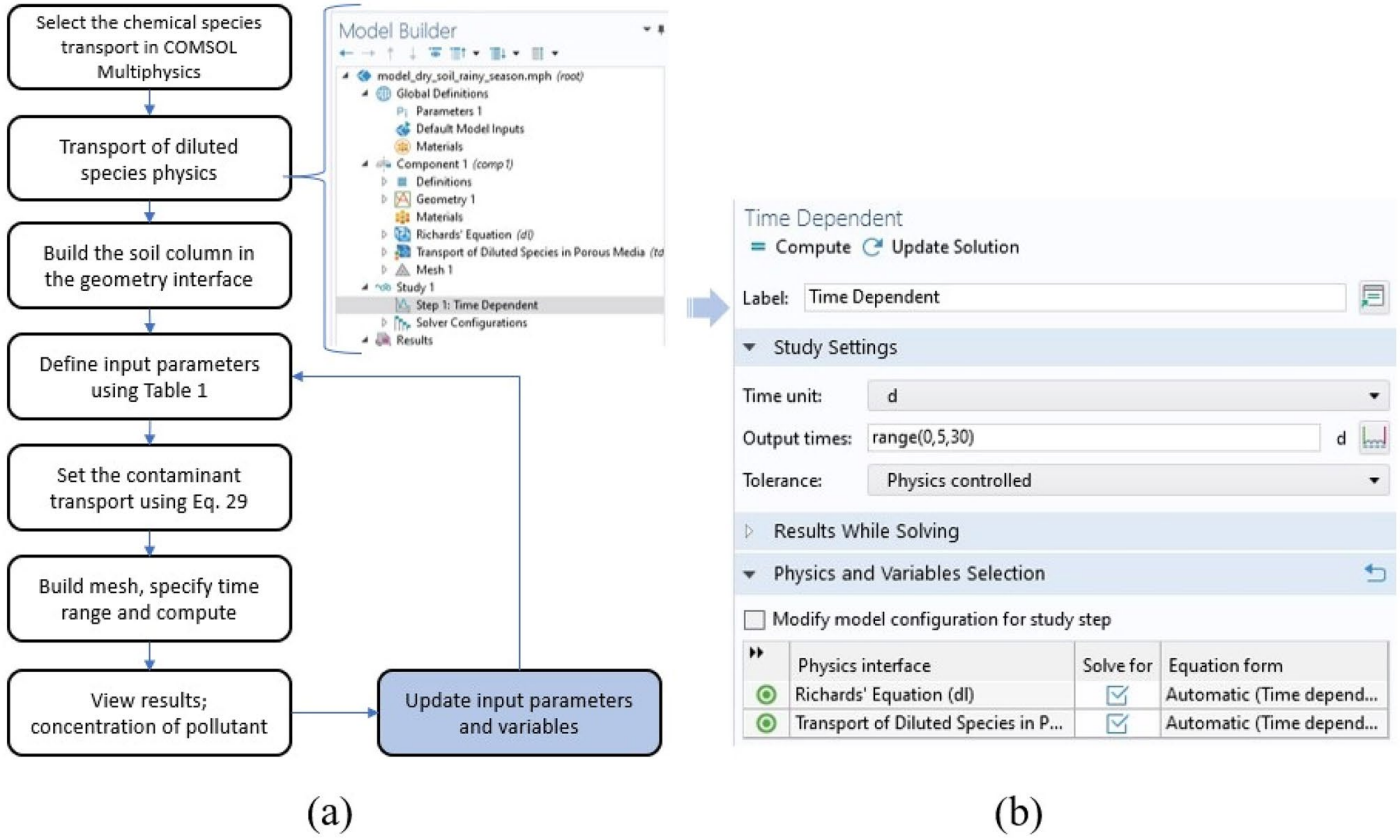
(**a**) Procedure used to implement the numerical model in COMSOL (**b**) screen shots of software interface.

Prediction of the concentration of heavy metal contaminants in the liquid phase were determined through the solution of Eq. (29) for all heavy metals where C_T_ is concentration of respective contaminants (mol L^−1^), t is the time of transport (m), Js is the total concentration of contaminant in the leachate (mol L^−1^), Z is the thickness of the soil column (cm) and  is the error term. The COMSOL Multiphysics software 6.0 was used to simulate the movement of pollutants in the soil^37,38^; first, geometry interface was used to design the model of the soil column, then, the transport of diluted species physics was used to create the starting point for the source of heavy metal pollution, and finally, the time-dependent study was used to simulate the change in pollutant concentration with time. The soil in the sampling area was characterized. The depth of the studied soil area is 90; 60 and 30 cm, heavy metal pollutants contained in leachate in the soil can be regarded as a solute in a homogeneous medium according to the law of migration^37,39^. Therefore, the model can be displayed as a plane. The entire design layout is shown in Fig. 3. The numerical simulation was carried in COMSOL Multiphysics software 6.0, using HP folio 1040 with a processing speed of 2.7 GHz, RAM 8 GB, intel core i5 at a computation time 50 min. The parameters used for heavy metal transport through soil with their respective units are shown in Table 3. Figure 3 depicts the procedure for simulation of numerical model built in COMSOL Multiphysics.

#### Statistical analysis

The data generated were analyzed using descriptive and inferential statistics. Descriptive statistics employed included mean, standard deviation and standard error of mean while inferential statistics used were two-way Analysis of Variance (ANOVA), Pearson’s Product Moment Correlation and Factor Analysis. Significantly different means were separated using the Duncan multiple range test at 5% level of probability^40–42^.

In addition, to evaluate the numerical model the accuracy and performance of the COMSOL Multiphysics prediction, the root means square error (RMSE) and the mean square error (MSE) were utilized as a function of error^43,44^ via Eqs. (30) and (31):

30 $$\mathrm{RMSE}=\sqrt{\frac{1}{\mathrm{N}}\sum_{\mathrm{j}=1}^{\mathrm{N}}{(\mathrm{Experimental}-\mathrm{Predicted})}^{2}}$$

31 $$\mathrm{MSE}=\frac{1}{\mathrm{N}}\sum_{\mathrm{j}=1}^{\mathrm{N}}{(\mathrm{Experimental}-\mathrm{Predicted})}^{2}$$

## Results and discussion

### Model analysis and validation

#### Leachate transport model

The volume of leachate transported over a given distance of the soil was modeled based on Eq. (17). Practical determination of the observed and estimated quantity of leachate (leachate flux) requires that the soil hydraulic conductivity (unsaturated) must be known. The real difficulty, therefore, was in finding K. In this study, unsaturated hydraulic conductivity was determined from transmissivity coefficient of the soil using Philip’s infiltration equation. Table 1 shows result of infiltration run conducted in the dumpsite soil.

**Table 1:** Infiltration characteristics of the dumpsite soil.

Parameter	I_o__(_cm min^−1^)	I_f_ (cm min^−1^)	S (cm min^−1^)	A (cm min^−1^)
JNPT01	0.40	0.10	0.575	2.173
JLPT01	0.30	0.09	0.513	2.257
AGPT01	0.50	0.08	0.711	2.376
Min	0.30	0.08	0.51	2.17
Max	0.50	0.10	0.71	2.38
Mean	**0.40**	**0.09**	**0.60**	**2.27**
Sd	0.08	0.01	0.08	0.08
Cv	20.41	9.07	13.79	3.67
JNPT02	0.30	0.08	0.591	2.292
JLPT02	0.20	0.07	0.634	2.229
AGPT02	0.80	0.15	0.749	2.463
Min	0.20	0.07	0.59	2.23
Max	0.80	0.15	0.75	2.46
Mean	**0.43**	**0.10**	**0.66**	**2.33**
Sd	0.26	0.04	0.07	0.10
Cv	60.57	35.6	10.14	4.25
JNPT03	0.40	0.07	0.650	2.308
JLPT03	0.30	0.11	0.592	1.820
AGPT03	0.60	0.20	0.770	2.231
Min	0.30	0.07	0.59	1.82
Max	0.60	0.20	0.77	2.31
Mean	**0.43**	**0.13**	**0.67**	**2.12**
Sd	0.12	0.05	0.07	0.21
Cv	28.78	42.92	11.05	10.11

JNPT01 = Point 1 in June; JLPT01 = Point 1 in July; AGPT01 = Point 1 in August, JNPT02 = Point 2 in June; JLPT02 = Point 2 in July; AGPT02 = Point 2 in August, JNPT03 = Point 3 in June; JLPT03 = Point 3 in July; AGPT03 = Point 3 in August.

Significant values are in [bold].

Infiltration characteristics of the dumpsite shows close similarities among the three points conducted. Generally, initial (Io) and final (I_f_) infiltration rate obtained in the study is low. Values of l_o_ ranged from 0.30 to 0.50 cm/min with mean of 0.40 ± 0.08 cm/min at PT01; 0.20–0.80 with mean of 0.43 ± 0.26 cm/min at PT02 and between 0.30 and 0.60 cm/min with mean of 0.43 ± 0.12 cm/min at PT03. All of them have their means between 0.40 and 0.43 cm/min. Similarly I_f_ ranged from 0.08 to 0.10 cm/min with mean of 0.09 ± 0.01 cm/min at PT01; 0.07–0.15 cm/min with mean of 0.10 ± 0.04 cm/min at PT02 while it was between 0.07and 0.20 cm/min with mean of 0.13 ± 0.05 cm/min at PT03. The means suggest low infiltration rate in the dumpsite soil.

Sorptivity and transmissivity are two sisters in relation to soil water flow. While sorptivity relates to absorption and adsorption of water in the soil matrix, transmissivity is the characteristics of the soil which indicates the contribution of the soil to intake of water arising from gravity. In relation to soil water potential, sorptivity arises from matric potential while transmissivity results from gravitational potential. Generally, matrix potential is always low and sometimes negligible as noted by Onofiok^45^ and Edem^31^. This can be seen experimentally in value of sorptivity of the soil which is low. Generally, values of sorptivity are lower than that of transmissivity in all the three points for the nine cases obtained in the study. It ranged from 0.510 to 0.710 cm min^−1^ at PT01 with mean of 0.60 ± 0.08 cm min^−1^, 0.59–0.75 cm min^−1^ with mean of 0.66 ± 0.07 cm min^−1^ at PT02 and between 0.59 and 0.77 with mean of 0.67 ± 0.07 cm min^−1^ at PT03. On average, mean sorptivity obtained in the study was 0.64 ± 0.09 cm min^−1^.

Similarly, values of transmissivity (A) obtained in the study are fairly high. It ranged from 2.17 to 2.38 with mean of 2.27 ± 0.08 cm min^−1^ at PT01, 2.23–2.46 with mean of 2.33 ± 0.10 cm min^−1^ at PT02 and 1.82–2.31 with mean of 2.12 ± 0.21 cm min^−1^ at PT03 with average of 2.24 ± 0.18 cm min^−1^. The average transmissivity of the soil revealed that A which represents the hydraulic conductivity of the soil is almost constantly distributed throughout the experiment across the three points. This could be attributed to several factors such as water content, total porosity, pore size distribution and pore continuity. It is reported that hydraulic conductivity increases with increasing moisture content in the soil. This is why saturated hydraulic conductivity is far greater than unsaturated hydraulic conductivity^45^. Hydraulic conductivity also increases with increasing total porosity. This is because, as total porosity increases, more pores or conducting spaces are available for water to be conducted. The converse is true as porosity decreases. For this reason, when the porosity of a soil is reduced, for instance by compaction, K decreases. More water is conducted through the larger (macro) pores than the meso or micro pores. The latter is used mainly for retention.

Thus, K of a soil that has predominantly large pores will be larger than that of the same soil when these large pores are lost, say through compaction. So changes in pore size distribution with depth contribute to decrease in hydraulic conductivity. Hence, hydraulic conductivity is higher when the pores are continuous and straight than when they are discontinuous and meandering ^8^. In a meandering pore water takes a longer time to reach its destination than when it is flowing through a straight pore. In a discontinuous pore water movement stops where the pore is blocked, then the water finds another pore to continue its movement. Consequently flow is reduced, hence reduction in flux with depth. In this study, the soil was unsaturated, hence, water content was the same and the porosity was similar across the three points and at the same depth resulting in constant K. This has affirmed Darcy’s law of steady state water flow that the flux is directly proportional to $\Delta \mathrm{H}/\Delta \mathrm{x}$ with K as constant of proportionality^32^.

**Figure 4:**
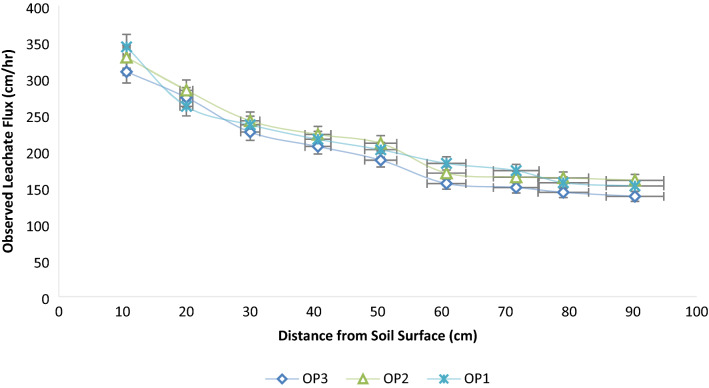
Observed leachate flux obtained in the study area.

Again, the higher transmissivity in the soil than sorptivity is an indication that the contribution of gravitational potential in leachate transport through the soil is significantly higher than the contribution of matric potential and this confirms why matric potential is often neglected unlike gravitational potential in soil water transport model. The result agrees perfectly with finding of Ogban^46^ in Uyo, Edem^31^ in Uyo and Onofiok^45^ in Nsukka who, in their different studies, reported higher transmissivity than sorptivity. Substituting values of transmissivity (A) for unsaturated conductivity (K) in Eq. (15), the soil water (leachate) flux was determined for various distances from soil surface (depth). This gives the observed flux values used in modeling leachate transport in the study area (Fig. 4).

#### Determination of best fit model

The observed leachate flux obtained from Eq. (15) were fitted into four regression functions namely linear function, Logarithmic, power and exponential functions to determine the best fit model for relationship between leachate flux and soil depth. Result shows that the power function is the best fit model in describing the relationship between distance and quantity of leachate entering the soil per unit time (Table 2). This is informed by its R^2^ value, residual and standard error of the estimate. For samples obtained from PT01, the power function had R^2^ of 0.996 higher than the Log, linear and exponential functions with 0.979, 0.808 and 0.897 respectively. For PT02 samples, the power function still recorded the highest R^2^ of 0.959 followed by Logarithmic function with 0.952 to exponential function with 0.878 while the linear Function had the least (0.798). Samples at PT03 still revealed that the power function is the leading model with R^2^ of 0.988 followed by Log function with 0.985 to exponential (R^2^ = 0.911) while the linear function had the least (R^2^ = 0.832). Hence, leachate transport model of Uyo Municipal solid waste dumpsite (LEATRAM) was established using the power model as expressed in Eq. (32):

**Table 2:** Regression parameters of various estimation functions.

Sampling point	Function	R^2^	Residual	SE	Best function
PT01	Linear	0.808	719.68	26.827	
	Logarithmic	0.979	77.435	8.8	
	Power	0.996	0.0	0.016	Power
	Exponential	0.897	0.007	0.083	
PT02	Linear	0.798	669.57	25.876	
	Logarithmic	0.952	159.98	12.648	
	Power	0.959	0.003	0.051	Power
	Exponential	0.878	0.008	0.088	
PT03	Linear	0.832	603.55	24.567	
	Logarithmic	0.985	52.484	7.245	
	Power	0.988	0.001	0.031	Power
	Exponential	0.911	0.007	0.085	
Average	Linear	0.840	645.625	25.409	
	Logarithmic	0.980	78.920	8.884	
	Power	0.999	0.001	0.029	Power
	Exponential	0.914	0.007	0.083	

*SE* standard error of the estimate.

32 $$\mathrm{q}=333.85{\mathrm{x}}^{-0.364}$$

**Figure 5:**
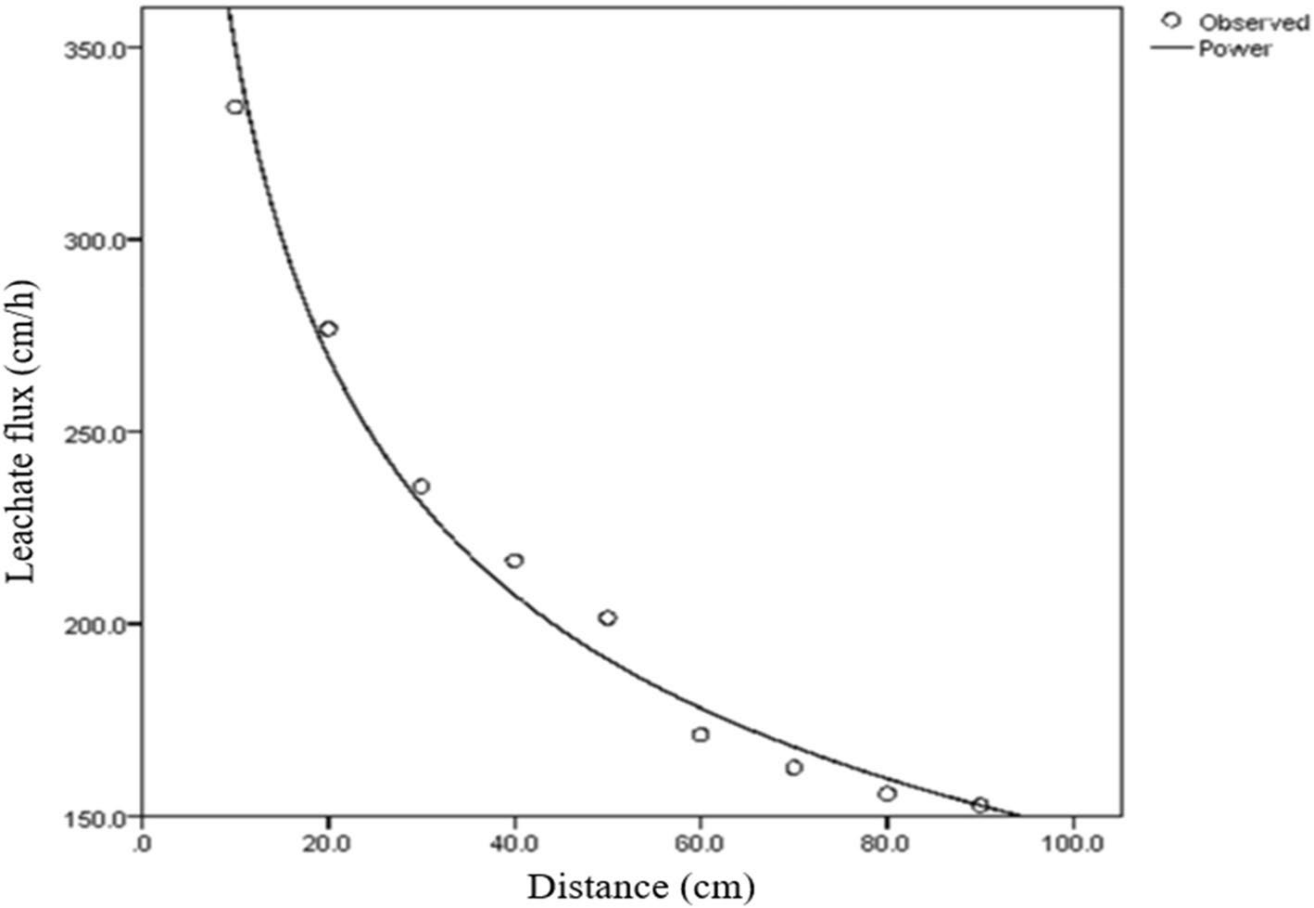
Leachate transport in dumpsite soil of Uyo.

The model reflects the general flow model originally proposed by Beven and German^47^ for macro pore flow in soils as macro pore flow law for channel flow in waste dumpsite, where q is the leachate flux density in cm/hr per cross sectional area of the soil, the constant 333.85 can be interpreted as the integral effect of the surface geometry and spatial characteristics of flow path called the sorptivity, x is the thickness of the soil representing depth of transport while the negative exponent represent the influence of soil structure called tortuosity, indicating that the flux is in the direction of decreasing hydraulic head as stated in the third principle of the model. The power function, identified as the best fit model for leachate transport in Uyo dumpsite soil revealed that rate of transport of leachate in soil of the study area slows down with depth (Fig. 5). This also suggests a reduction in the driving force ($\Delta \mathrm{H}/\Delta \mathrm{x}$) and distance variability. Initially, when the soil is dry, the driving force is very high and flow is therefore high. With time, ($\Delta \mathrm{H}/\Delta \mathrm{x}$) gets smaller and flow decreases as depth of wetting increasing. This follows from the fact that as depth of wetting increases, $\Delta \mathrm{x}$ will also increase while $\Delta \mathrm{H}$ remains constant. Hence, the quotient $\Delta \mathrm{H}/\Delta \mathrm{x}$ decreases resulting in decrease in q.

The result also shows that the flux is changing with soil depth indicating that water is stored. Thus, the flux entering the soil would not equal the flux leaving it under unsaturated condition. The difference between what is entering and what is leaving is storage; storage can be expressed as a change in volume water content with time ($\mathrm{\delta \theta v}/\mathrm{\delta t})$ and the difference between inflow and outflow can be expressed as the change in flux along the length of the soil column ($\mathrm{\delta q}/\mathrm{\delta x}$), where $\theta v$ is the volumetric water content of the soil, q the flux transporting through a unit area of the soil at a given time while x is the thickness of the soil or distance of transport from the soil surface. The description given above is termed conservation of mass which states that water is not lost or destroyed: what flows in is either stored or f1ows out from the soil column^48^.

### Contaminant transport model

Leachate entering the soil also carries along various contaminants at varied concentration. In this study, only the heavy metal contaminants were modeled. Leachate flux obtained at respective depth was fitted into contaminant transport Eq. (28) to obtain the estimated values of contaminants. Tables 3 and 4 showed the observed and estimated concentration of contaminants. Result obtained showed a direct relationship between concentration of contaminant and leachate flux. Both revealed a decreasing trend in the concentration of the contaminants as distance from soil surface (depth) increases. This suggests that there is adsorption of the contaminants to the soil surface. It is known that the soil matrix contains negatively charged ions which help in retention of the basic cations in the soil for plant use^35^. The result showed that some of these metals are being stored as they leached down the soil. As explained in leachate transport, that in non-transient flow, leachate is stored while some is transported along the soil column. Part of the storage contains certain quantity of various contaminants present in the leachate resulting in decrease in concentration of both the leachate and contaminants as depth of the soil increases.

**Table 3:** Observed concentration of the contaminants in the soils (mol L^−1^).

Distance (cm)	Fe	Cu	Zn	Pb	Mn	Ni	Cd	
10	0.0694	0.0228	0.0343	0.0597	0.0373	0.0483	0.0590	
20	0.0361	0.0216	0.0125	0.0273	0.0227	0.0268	0.0259	
30	0.0260	0.0116	0.0018	0.0115	0.0133	0.0139	0.0240	
40	0.0148	0.0028	0.0014	0.0031	0.0027	0.0034	0.0128	
50	0.0036	0.0008	0.0008	0.0020	0.0018	0.0034	0.0021	
60	0.0036	0.0005	0.0008	0.0011	0.0019	0.0029	0.0019	
70	0.0030	0.0002	0.0008	0.0012	0.0011	0.0019	0.0014	
80	0.0024	0.0001	0.0008	0.0009	0.0001	0.002	0.0016	
90	0.0024	0.0001	0.0006	0.0008	0.0001	0.002	0.0007

**Table 4:** Estimated concentration of the contaminants from leachate flux (mol L^−1^).

Distance (cm)	Fe	Cu	Zn	Pb	Mn	Ni	Cd
10	0.094	0.033	0.055	0.124	0.078	0.106	0.115
20	0.040	0.014	0.020	0.045	0.026	0.035	0.041
30	0.021	0.006	0.009	0.022	0.015	0.023	0.015
40	0.014	0.004	0.006	0.013	0.010	0.018	0.013
50	0.010	0.004	0.006	0.012	0.007	0.014	0.009
60	0.009	0.002	0.004	0.008	0.004	0.007	0.004
70	0.007	0.002	0.003	0.011	0.004	0.006	0.004
80	0.006	0.001	0.002	0.007	0.003	0.006	0.003
90	0.006	0.001	0.001	0.005	0.003	0.005	0.003

### Model validation

**Figure 6:**
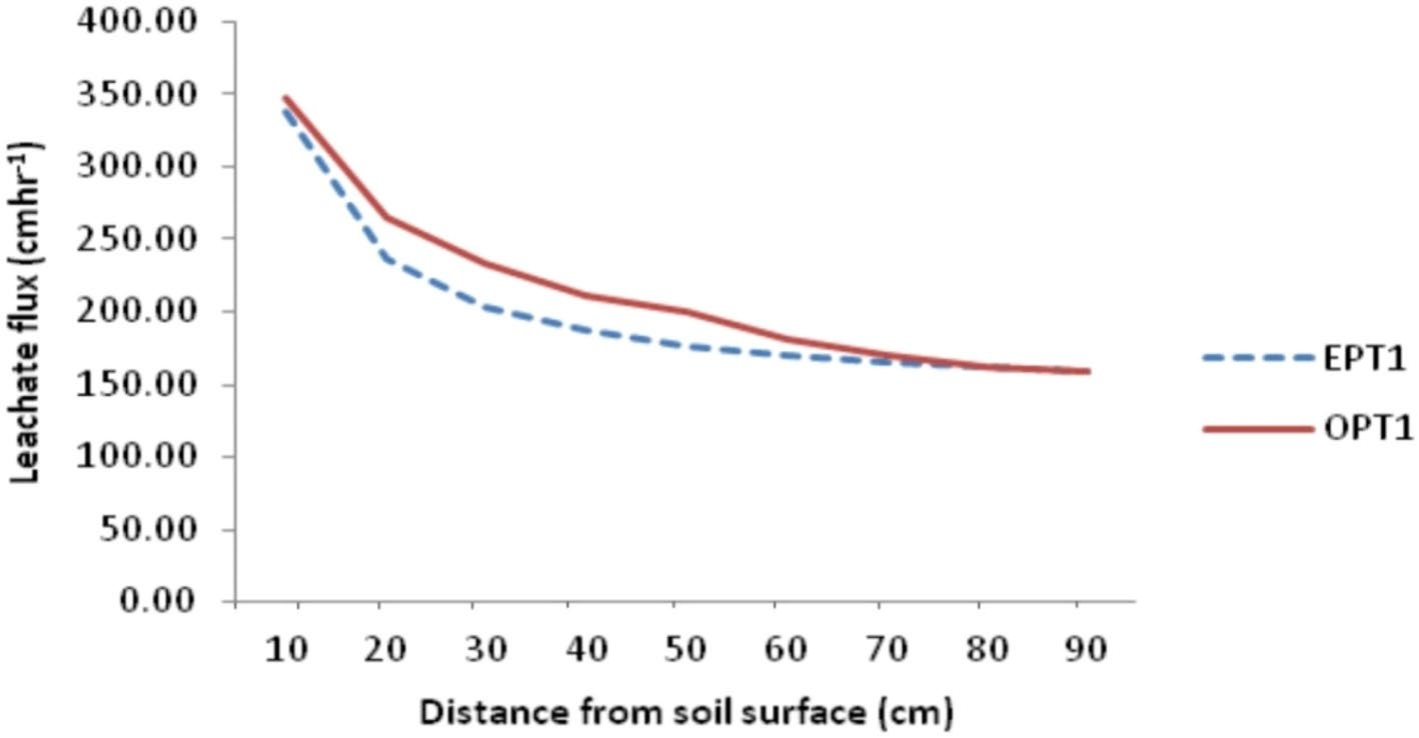
Pattern of observed and estimated leachate flux at PT01 in the dumpsite soil.

**Figure 7:**
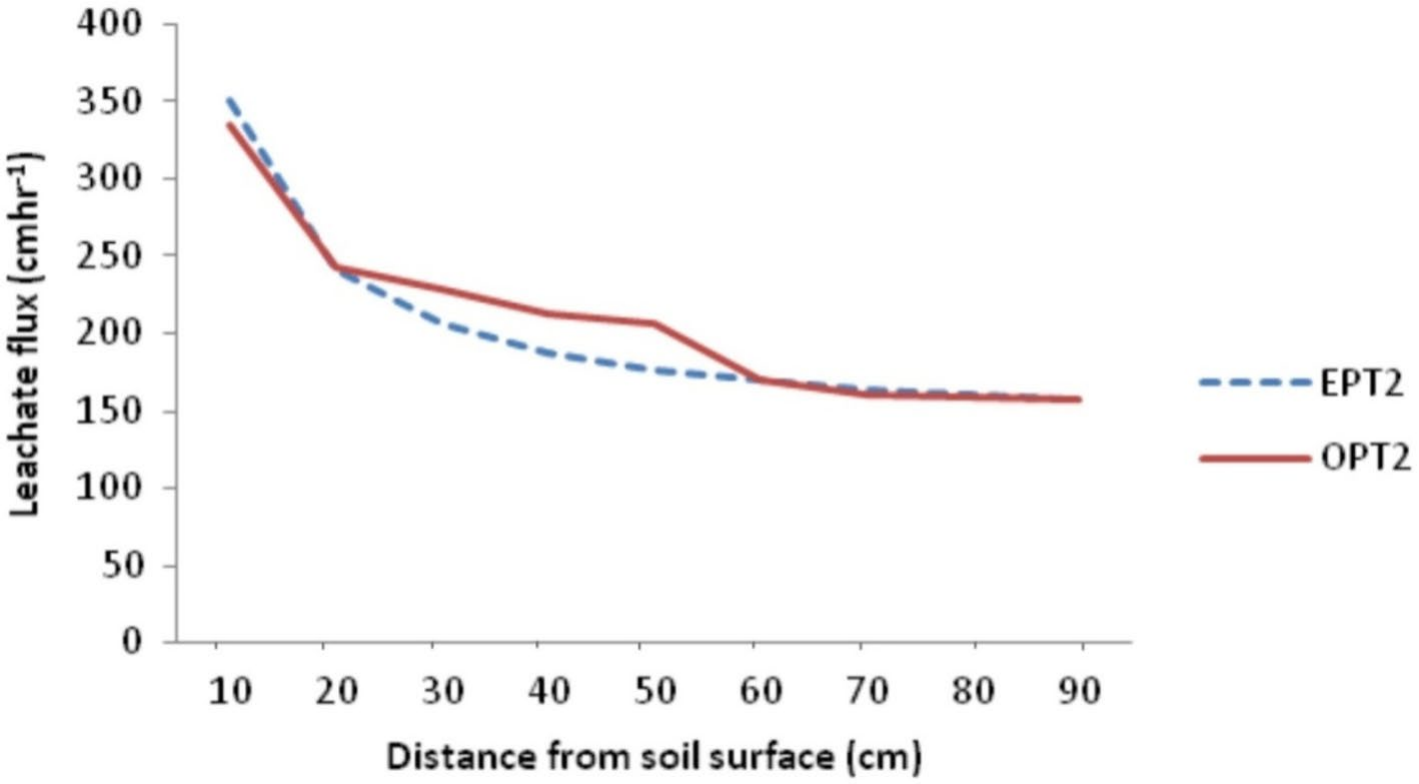
Pattern of observed and estimated leachate flux at PT02 in the dumpsite soil.

**Figure 8:**
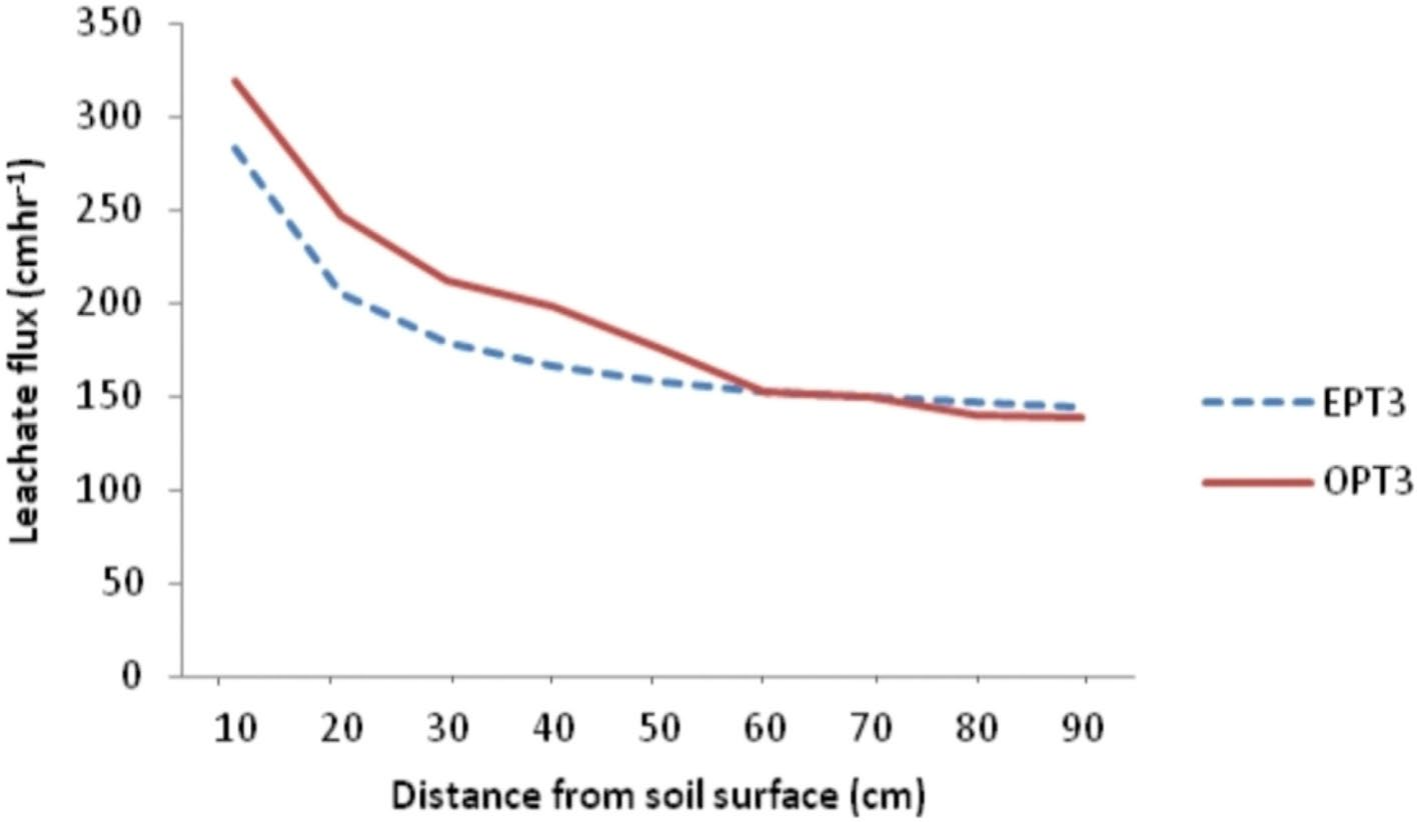
Pattern of observed and estimated leachate flux at PT03 in the dumpsite soil.

**Figure 9:**
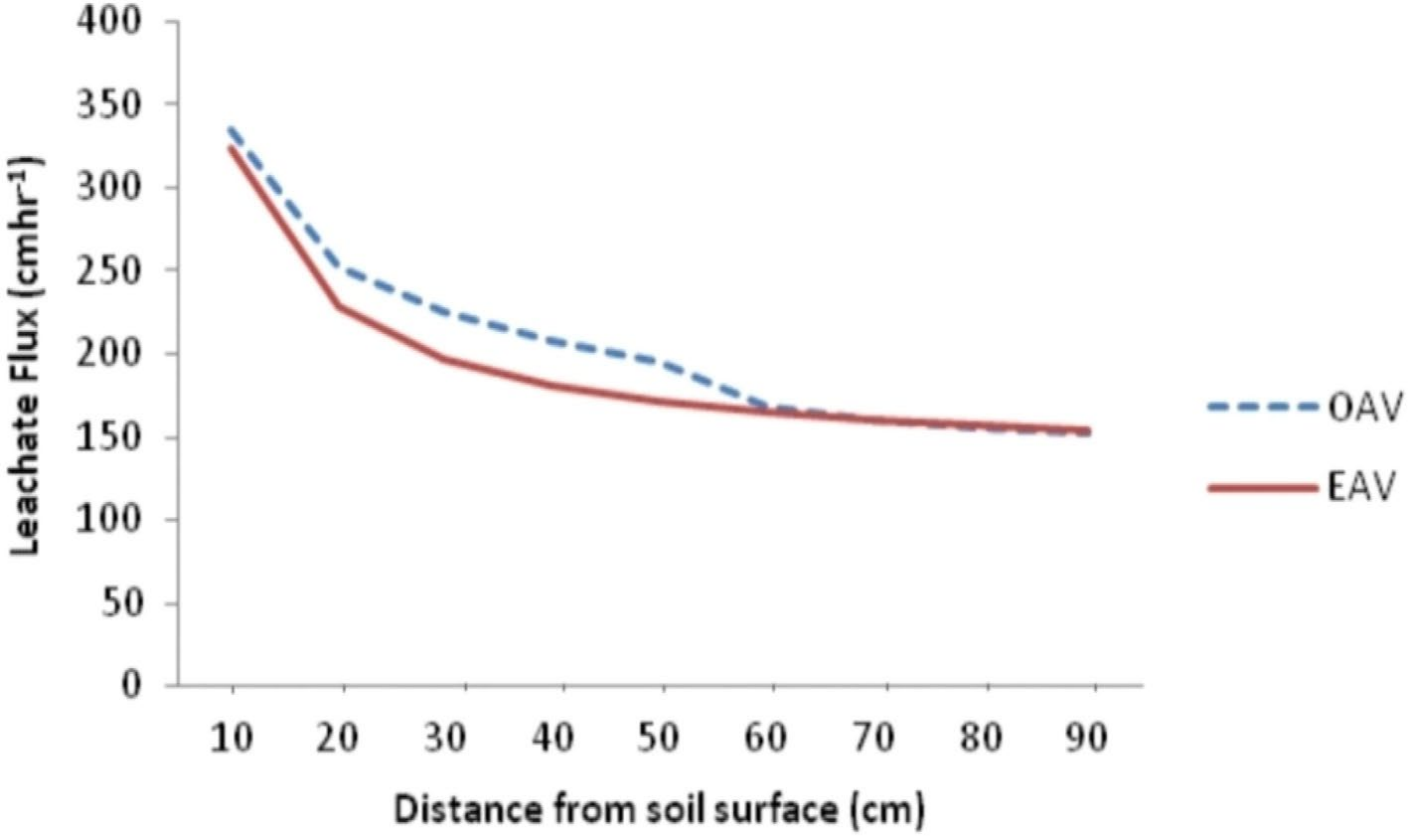
Pattern of observed and estimated leachate flux for average leachate flux in the dumpsite soil.

Leachate transport model obtained in this study was validated by subjecting the observed and estimated leachate flux from the three sample groups into power regression equation to determine the extent to which the observed values can predict the estimated values from the model. Result shows that the observed leachate flux predicted the estimated leachate flux by 96.40, 93.90 and 94.40 for samples from PT01, PT02 and PT03 respectively with residuals of 134.180, 265.82 and 126.68 and standard error (SE) of 11.58, 16.30 and 11.26 respectively (Table 5). Figures 6, 7, 8 and 9 show the observed and estimated leachate flux at PT01, PT02, PT03 and the average. Among the contaminant, observed and estimated values showed significant agreement but with lower R^2^ than that of leachate except Zn. From the results, Fe predicted the estimated values from the designated depths by 87.10 while Cu, Zn, Pb, Mn, Ni, and Cd predicted their estimated counterparts by 84.80, 94.10, 82.60, 86.40, 82.00 and 89.70% respectively^49^.

**Table 5:** Regression of observed and estimated leachate flux and contaminants.

Parameter	R^2^	Residual	SE	Model equation
Leachate position				
PT01	0.964	134.180	11.583	${\mathrm{q}}_{\mathrm{L}}=346.14{\mathrm{x}}^{-0.358}$
PT02	0.939	265.820	16.300	${\mathrm{q}}_{\mathrm{L}}=330.48{\mathrm{x}}^{-0.344}$
PT03	0.944	126.684	11.255	${\mathrm{q}}_{\mathrm{L}}=262.39{\mathrm{x}}^{-0.296}$
Average	0.977	157.003	12.530	${\mathrm{q}}_{\mathrm{L}}=333.85{\mathrm{x}}^{-0.364}$
General equation				${\mathrm{q}}_{\mathrm{L}}={\mathrm{Cx}}^{-\mathrm{n}}$
Contaminants				
Fe	0.891	0.0001	0.011	${q}_{Fe}=0.102{x}^{-1.754}$
Cu	0.848	0.0001	0.004	${q}_{Cu}=0.081{x}^{-2.889}$
Zn	0.941	0.0001	0.004	${q}_{Zn}=0.028{x}^{-1.898}$
Pb	0.826	0.0001	0.017	${q}_{Pb}=0.112{x}^{-1.380}$
Mn	0.864	0.0001	0.009	${q}_{Mn}=0.078{x}^{-1.536}$
Ni	0.820	0.0001	0.014	${q}_{Ni}=0.104{x}^{-1.395}$
Cd	0.897	0.0001	0.012	${q}_{Cd}=0.121{x}^{-1.741}$

NB: SE is the Standard error of the estimate; R^2^ is the coefficient of determination; ${q}_{L}$ is the leachate flux; C is the transmissivity coefficient; $x$ is the thickness of the soil; n is the nonlinear exponent.

**Figure 10:**
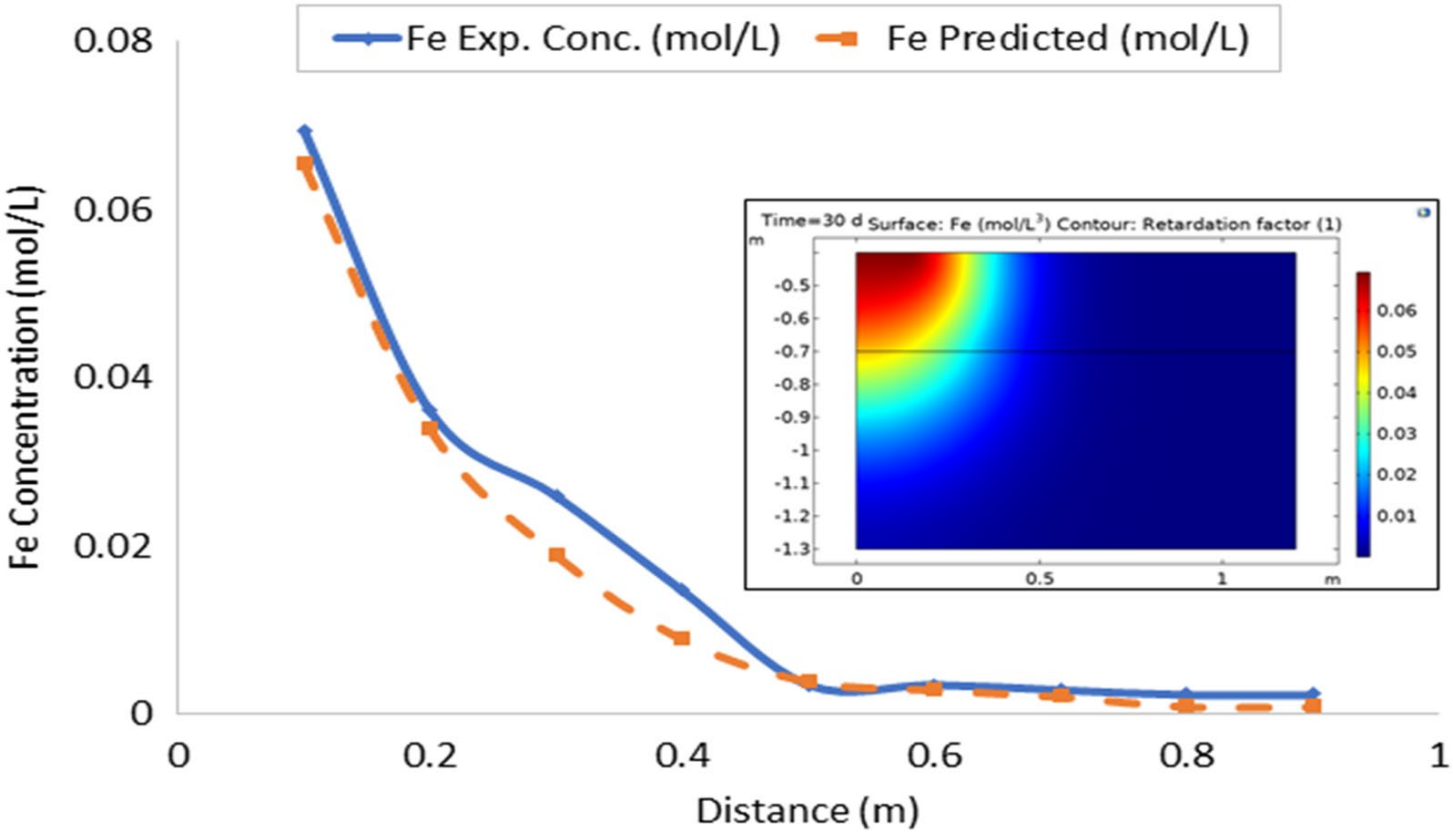
Fe transport in dumpsite soil of the study area.

**Figure 11:**
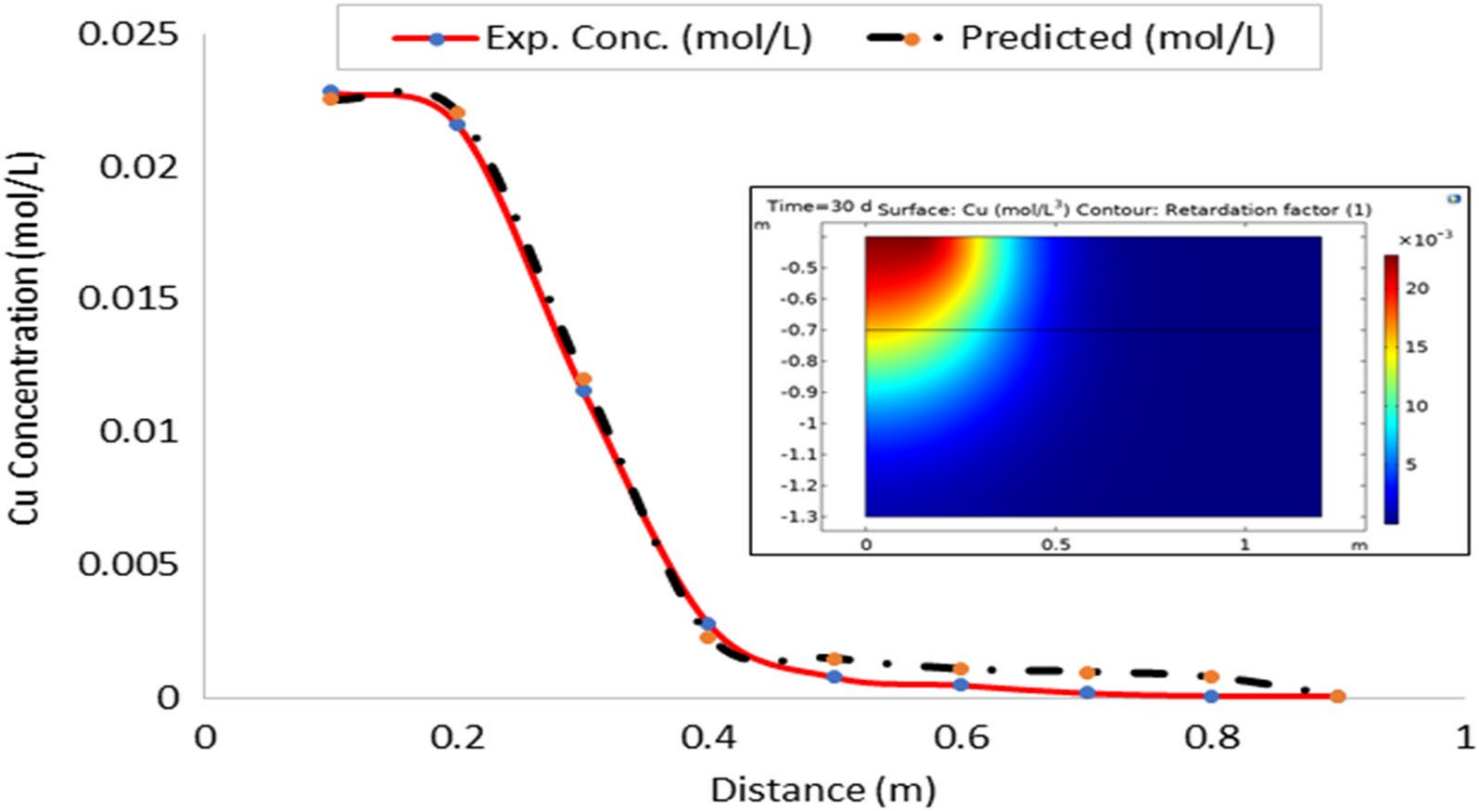
Cu transport in dumpsite soil of the study area.

**Figure 12:**
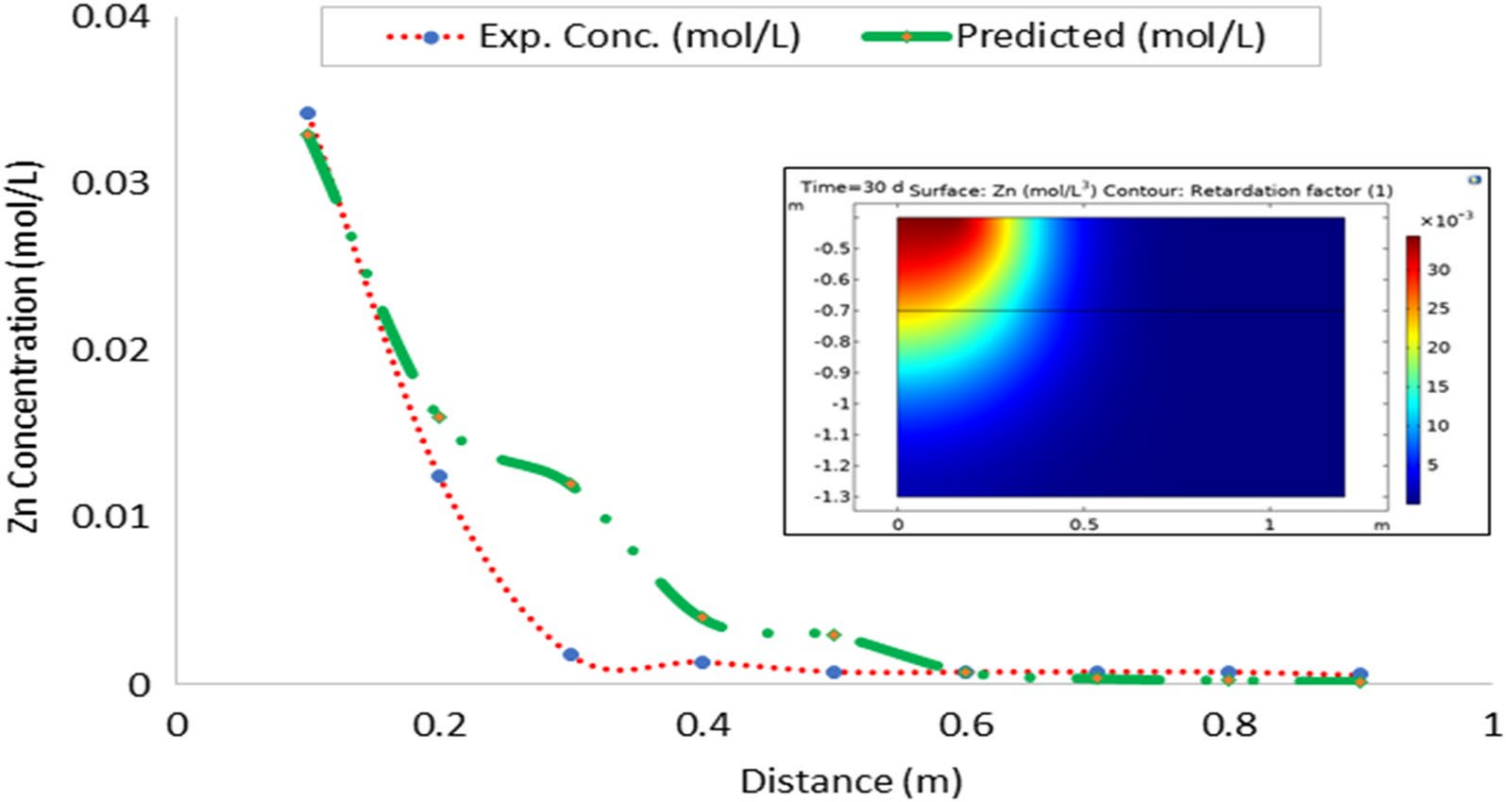
Zn transport in dumpsite soil of the study area.

**Figure 13:**
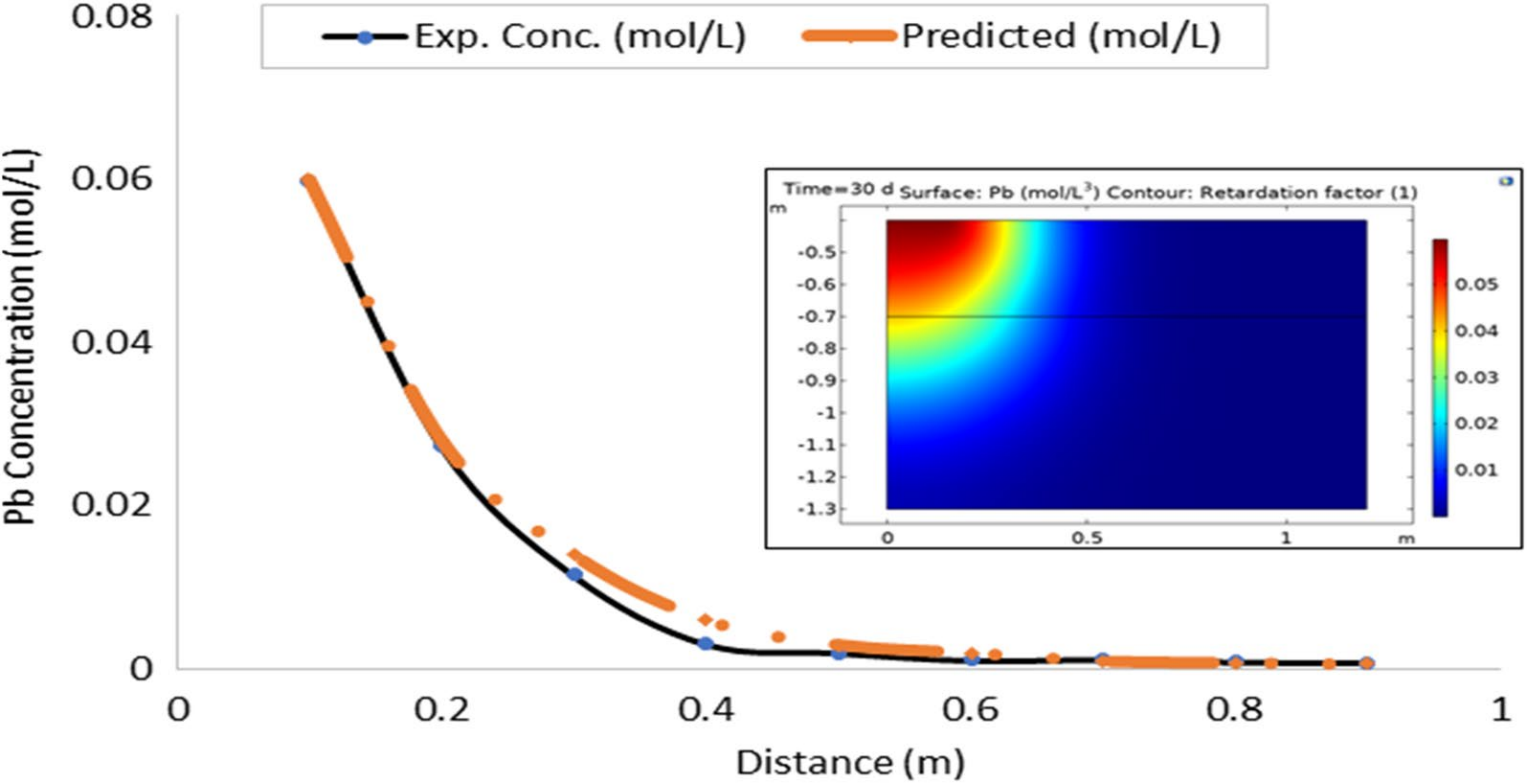
Pb transport in dumpsite soil of the study area.

**Figure 14:**
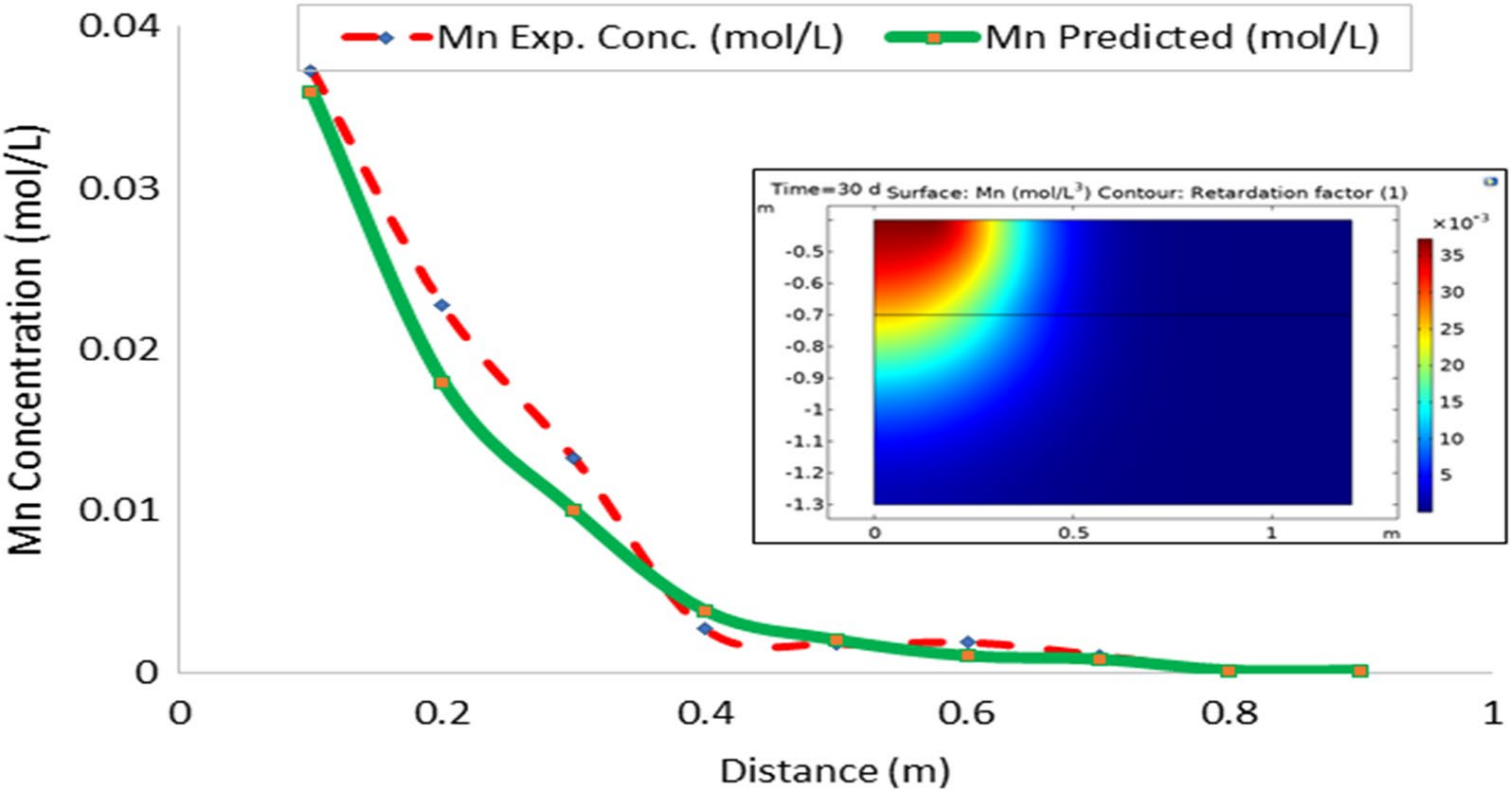
Mn transport in dumpsite soil of the study area.

**Figure 15:**
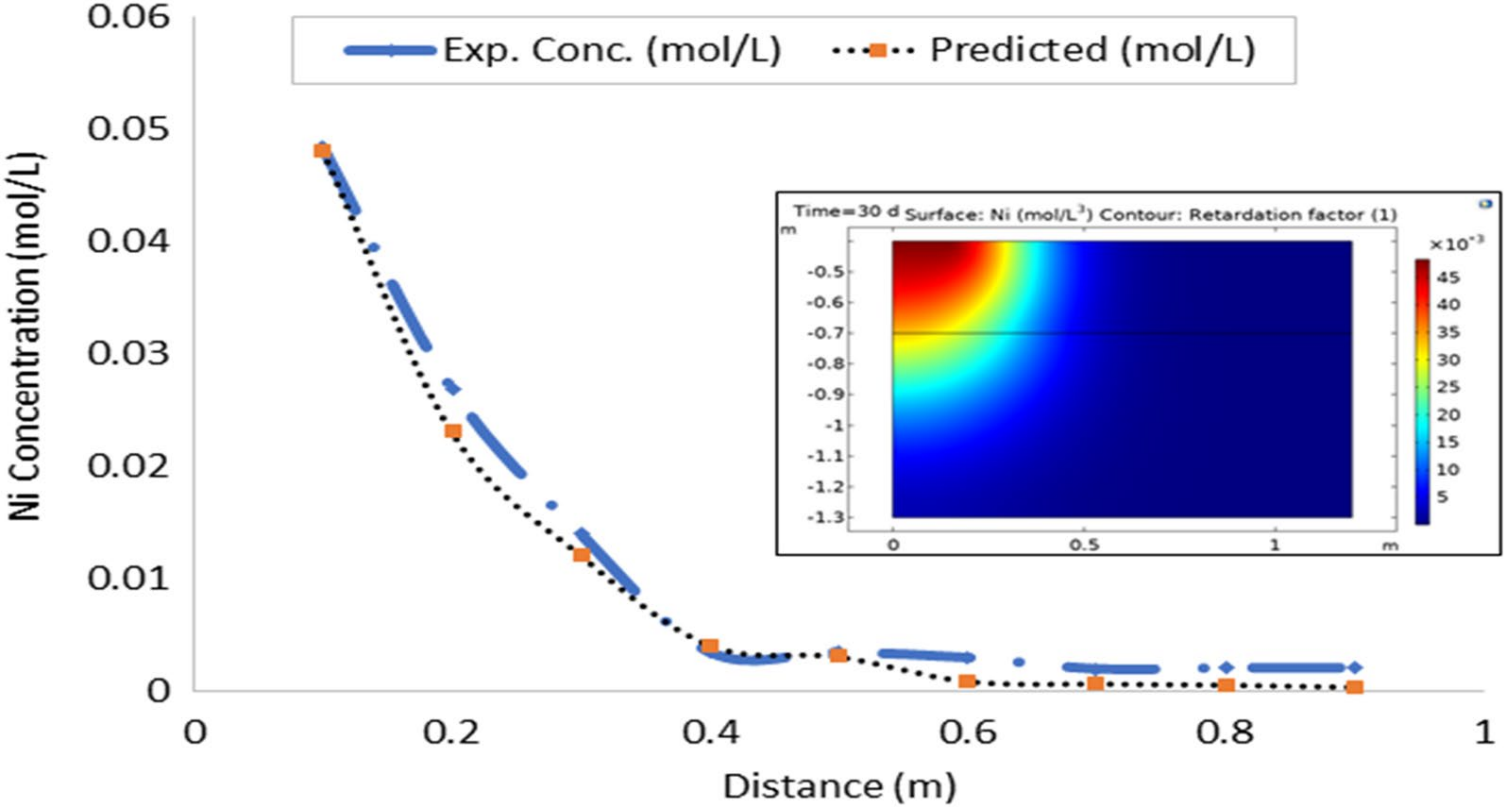
Ni transport in dumpsite soil of the study area.

**Figure 16:**
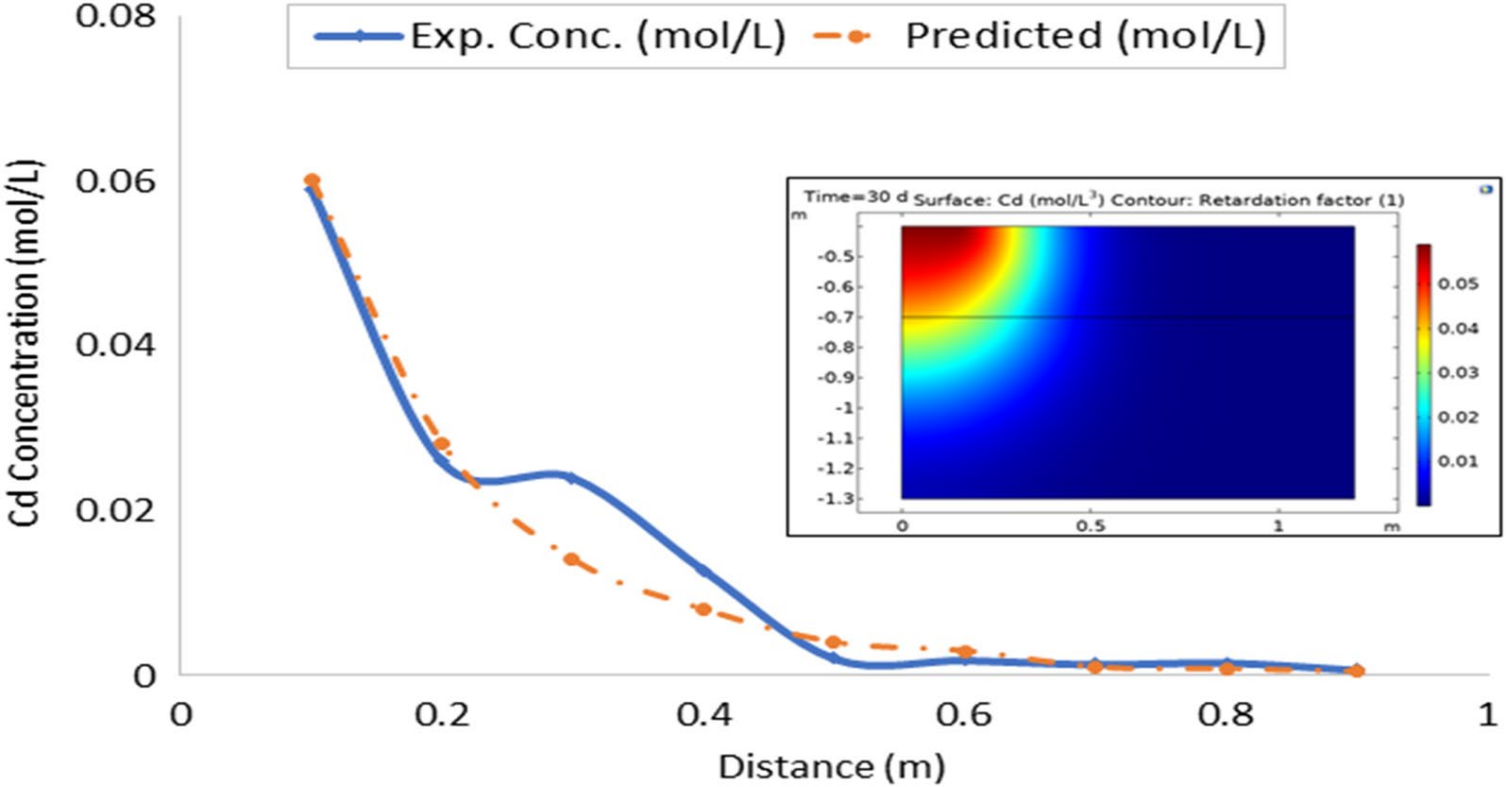
Cd transport in dumpsite soil of the study area.

On the whole, there is a strong agreement between the observed and estimated leachate flux and concentration of contaminants from the developed leachate and contaminant transport models in the study area. Figures 10, 11, 12, 13, 14, 15 and 16 show transport of various contaminants in dumpsite soil of the study area. Their model equation revealed that the rate at which each of the contaminant is transported across depth in the dumpsite soil is very low and may not be continuous unlike leachate transport which is continuous and at very high rate. However, both leachate and contaminant transport along the soil column followed the same model but different coefficient and rate. This could be attributed to diffusion and dispersion effect of the contaminants unlike the leachate. Both models can be utilized for various estimations within 0–1 m depth of the soil. Therefore, it is widely recommended for Uyo soil and also in locations with similar physical, chemical and hydrological properties of the soil^50^.

To compare the migration of pollutants over time in a given area, pollution points were selected in the soil column direction from the simulation diagram (geometry). The horizontal distance from the pollution source to the current location is displayed in the positive vertical direction, and the concentration changes with time. The flow field is the same, and the main direction of transport is the vertical direction. The concentration distributions of Fe, Pb, Cu, Mn, Ni, Zn and Cd after 30 days period of infiltration are shown in Figs. 9, 10, 11, 12, 13, 14 and 15. The pollutants clearly have reached steady-state conditions, and it is expected that the heavy metals in the soil will contaminate a large soil area for a long time. Through the soil column simulation model, the simulation was carried using the initial concentrations of the selected heavy metals^51^. The experimental result of the selected heavy metals is combined with the COMSOL Multiphysics software simulation results as shown in Figs. 8, 9, 10, 11, 12, 13 and 14, it can be seen that regarding the migration of heavy metal pollutants in soil over time, with increasing soil distance, the seepage flow increases with a peak shape in the 2D plot at the top right corner of the graphs. According to the change in color, the concentration gradually decreases from top to bottom; dark blue indicates a lower concentration, and red indicates the maximum concentration. The migration of heavy metal pollutants mainly occurs in the distance range of 1–90 cm (0.1–0.9 m), and their contents are mainly concentrated in this interval. The soil surface was used to measure the total contents of the heavy metals Fe, Pb, Zn, Mn, Ni, Cd, and Cu, as well as the contents of the residual state of heavy metals. The results show that the concentration of each form of Fe, Pb and Cu in the municipal solid waste dumpsite area reached a high degree of pollution. It was observed that the findings of this work agree with that of Xie et al.^52^ who studied Cu and Cd transport in metal mining area.

However, the summary of error computation for validation of the numerical model from COMSOL Multiphysics is presented in Table 6.

**Table 6:** Summary of error computation for model validation.

Heavy metals	R^2^	MSE	RMSE
Fe	0.99484	0.00001201	0.00347
Cu	0.99889	0.00000029	0.00054
Pb	0.99836	0.00000190	0.00138
Mn	0.99236	0.00000409	0.00202
Ni	0.99690	0.00003322	0.00576
Zn	0.94906	0.00001446	0.00380
Cd	0.98001	0.00001490	0.00386

## Conclusion

The study showed that leachate and contaminants (heavy metals) transport in soil of the study area is in the power functional form. Their model equations revealed that the rate at which various contaminants is transported across depth in the dumpsite soil is very low and may not be continuous but leachate transport is continuous with a very high rate. Validation equation showed that the observed leachate flux predicted the estimated ones perfectly with very high R^2^ of over 95% justifying the reliability of the model in estimating leachate and contaminants transport in soil of the study area. This study provides a new insight on how the leachate and chemicals from Uyo MSW dumpsite are transported through the soil and causing pollution. The extent of pollution of the soil within Uyo dumpsite can be accurately predicted using leachate transport model of this study. This is one of the main contributions of this study to knowledge which can be used to clearly establish the influence of chemicals from the leachate of MSW dumpsite on the properties of soil for possible control measures to reduce the extent of soil pollution. Also, the study has given an update on the high pollution status of the soil by MSW leachate. Findings from the study has identified level in depth to which leachate from the dumpsite extends; this is highly beneficial to farmers and town planners for agricultural and environmental management policies. The finding has also cleared doubt and fear among the existing borehole owners on the quality of their borehole water due to continuous accumulation of waste in the dumpsite.

## Data Availability

All data generated or analysed during this study are included in this published article.
